# Perspective: Framework for Developing Prediction Equations for Estimating the Absorption and Bioavailability of Nutrients from Foods

**DOI:** 10.1016/j.advnut.2025.100481

**Published:** 2025-07-17

**Authors:** Connie Weaver, Seth Armah, Richard S Bruno, Andrew Fletcher, Raymond Glahn, Isabelle Herter-Aeberli, Tasija Karosas, Cornelia U Loechl, Veronica Lopez-Teros, Michael I McBurney, Alida Melse-Boonstra, Rachel Novotny, Manju B Reddy, Jessica Rigutto-Farebrother, Sherry Tanumihardjo, Emorn Udomkesmalee, Ellen Van Den Heuvel, Taylor Wallace, Pattanee Winichagoon

**Affiliations:** 1 School of Exercise and Nutritional Sciences, San Diego State University, San Diego, CA, United States; 2 Department of Nutrition, University of North Carolina at Greensboro, Greensboro, NC, United States; 3 Human Nutrition Program, The Ohio State University, Columbus, OH, United States; 4 Sustainable Nutrition Initiative, Riddet Institute, Massey University, Palmerston North, New Zealand; 5 Fonterra Research and Development Centre, Palmerston North, New Zealand; 6 Untied States Department of Agriculture—Agriculture Research Services (USDA-ARS), Robert W. Holley Center for Agriculture and Health, Ithaca, NY, United States; 7 Laboratory of Nutrition and Metabolic Epigenetics, Institute of Food, Nutrition and Health, ETH Zürich, Zürich, Switzerland; 8 The International Life Sciences Institute, Washington, DC, United States; 9 Nutritional and Health-related Environmental Studies Section, Division of Human Health, Department of Nuclear Sciences and Applications, International Atomic Energy Agency, Vienna International Centre, Vienna, Austria; 10 Department of Human Health and Nutritional Sciences, University of Guelph, Guelph, ON, Canada; 11 Gerald J. and Dorothy R. Friedman School of Nutrition Science and Policy, Tufts University, Boston, MA, United States; 12 Division of Human Nutrition and Health, Wageningen University & Research, Wageningen, The Netherlands; 13 Department of Human Nutrition, Food, and Animal Sciences, University of Hawai’i at Manoa, Honolulu, United States; 14 Department of Food Science and Human Nutrition, Iowa State University, Ames, IA, United States; 15 Global Center for the Development of the Whole Child, University of Notre Dame, Notre Dame, IN, United States; 16 Department of Nutritional Sciences, University of Wisconsin-Madison, Madison, WI, United States; 17 Institute of Nutrition, Mahidol University, Salaya, Nakhon Pathom, Thailand; 18 LLN NutriResearch, Eindhoven, The Netherlands; 19 Think Healthy Group, LLC, Washington, DC, United States; 20 School of Medicine and Health Sciences, George Washington University, Washington, DC, United States

**Keywords:** bioavailability, prediction equation, micronutrients, nutrient absorption, nutrients requirements

## Abstract

Current nutrient intake recommendations, nutritional assessments, and food labeling rely on estimated total nutrient content in foods and dietary supplements. However, the adequacy of nutrient intake depends not only on the total amount consumed but also on the fraction absorbed and utilized by the body. Accurate assessments of nutrient bioavailability require predictive equations or algorithms. This paper outlines a 4-step framework designed to guide researchers in developing such equations. The framework includes: *1*) identifying key factors that influence nutrient or bioactive compound bioavailability; *2*) conducting a comprehensive literature review of high-quality human studies to inform the development of predictive equations; *3*) constructing predictive equations based on these insights; and *4*) validate the equation, when feasible, to potentiate translation. This structured approach aims to enhance the accuracy and precision of nutrient bioavailability estimates, address data limitations, and highlight evidence gaps to inform future research and policy on nutrients and bioactive compounds.

**Statement of significance**

This paper is the first of its kind to present a stepwise methodology for developing bioavailability algorithms or predictive equations that do not account for host-specific factors, offering a foundational guide for future research and applications in nutrient bioavailability assessment.

## Introduction

**FIGURE 1:**
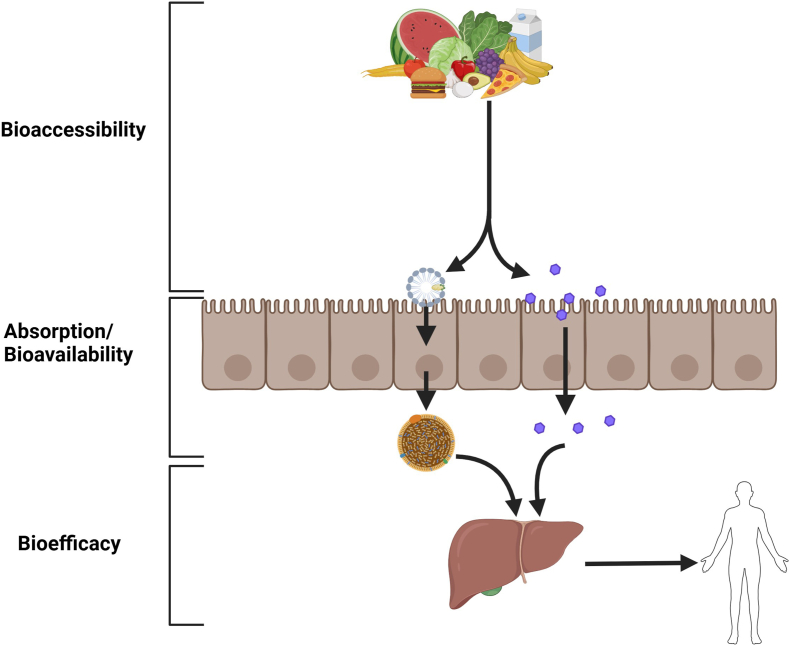
This figure illustrates various steps in digestion and metabolism for assessing the nutritional contribution of a food for a particular nutrient or bioactive compound.

Nutrient requirements and guidance are currently based on the total estimated amount of nutrients in foods (and sometimes supplements). These estimates are used for setting nutrient requirements, nutrition assessment, and food labeling for individuals and populations [1]. Total nutrient content determined analytically can lead to over- or underestimation of a food, supplement, or diet’s true nutritional value, due to reduced or enhanced nutrient absorption and/or utilization [2,3]. There are many potential steps involved as a nutrient or bioactive compound is consumed for it to ultimately be incorporated into a tissue or to be used functionally, as briefly illustrated in Figure 1. First, the food or supplement containing the compound of interest is masticated, reducing particle size, and is freed wholly or partially from the matrix. Then it is absorbed across the intestinal membrane, and for some substances, subsequent bioconversion postabsorption influences activity. Postabsorption bioefficacy can be greatly altered for some vitamins, such as vitamins D and E, and for bioactive compounds such as polyphenolics. Definitions for the processes involved are given in Box 1.

Box 1 **Definitions for processes involved in digestion and absorption.**

**Bioaccessible** refers to the amount (or fraction) of a nutrient that can be freed from the food matrix for absorption.

**Absorption** is the movement of a nutrient into systemic circulation.

**Predicted Bioavailability** is the extent to which absorption occurs. In other word, bioavailability is the fraction of the administered nutrient that reaches the systemic circulation in the unchanged form.

**Bioefficacy** considers the proportion of nutrient of bioactive that is converted to an active form in the body.

Alt-text: Box 1

Many nutrients and bioactive compounds have been studied for factors that influence absorption, which can be intrinsic or extrinsic. Intrinsic or host-specific factors include sex, age or life stage, physical activity, health status, nutritional status, the gut microbiome, genetics, and drug–nutrient interactions [4,5]. The principal determinant of absorption extrinsic to the consumer is the nutrient load in that food, for example, per unit or per serving of raw fresh, frozen, or cooked food, and whether the load exceeds the absorptive capacity of the gut. Indeed, availability of food composition data on the exact food consumed (e.g. species, variety, growing conditions, etc.), and in the form consumed (e.g. boiled, grilled, etc.), is the first challenge in obtaining accurate assessment of human nutrient absorption, but is beyond the scope of this paper [6]. Here, we focus on human nutrient absorption which may be influenced by the chemical structure and physical form of the nutrient, the physical form and composition of the food (food matrix) or supplement, which may contain absorption enhancers or inhibitors, interactions within the food matrix or meal, all of which may be influenced by preparation and processing [7,8]. For enriched or fortified foods and supplements, additional considerations are the chemical structure and physical form of added nutrients and microencapsulation of any nutrient that is sensitive to heat, light, and oxygen to which it may be exposed during processing and/or storage. With respect to supplements, considerations should also include the use of enteric coatings to bypass digestive processes, thereby helping deliver nutrients or bioactive compounds to specific regions of the gastrointestinal tract. These concepts apply to macronutrients, micronutrients, and bioactive compounds.

**FIGURE 2:**
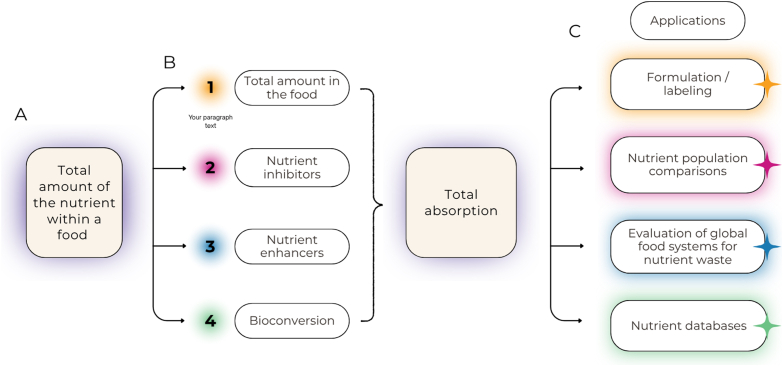
A sequential concept map illustrating the key factors involved in absorption or bioavailability and the application of relevant predictive equations. The map begins with (A) the total amount of nutrient ingested, (B) the critical factors to consider (or adjust for) when calculating total absorption or bioavailability, and (C) the various applications in which the predictive equation can be applied.

This paper will describe a framework for developing prediction equations for the absorption or bioavailability of individual nutrients or bioactive compounds. The framework includes step-by-step construction of a bioavailability or absorption prediction equation, identifies the information needed for its development, and considers confounding factors (Figure 2). The resulting consensus framework reported here is intended to guide future development of predictive absorption or bioavailability equations, and to determine data limitations and identify evidence gaps to direct future research for specific nutrients and bioactive compounds.

## Rationale and Approach

There is overwhelming evidence that many individuals in various populations have nutrient intakes below recommended levels, even before accounting for bioavailability [9]. For example, a modeling approach known as the DELTA model was used to estimate global and regional nutrient availability based on FAO’s food balance sheets and weighted distributions of food availability for estimating nutrient intake needs [10]. Projected nutrient gaps by 2030 identified shortfalls in calcium, vitamin E, iron, potassium, riboflavin, vitamin A, and vitamin B12, among other nutrients. Regions are unequally affected, and the distribution of food within a region is unequal, exacerbating the shortfalls within regions. Exercises such as the DELTA model illustrate inadequacies of some nutrients based on the total nutrient content, even before bioavailability of nutrients is considered.

To determine the absorption or bioavailability of a nutrient or bioactive compound from a food, meal, or diet, prediction equations can be developed so that every food and supplement does not have to be tested individually, which would be unfeasible. Nutrient absorption and bioavailability prediction equations have been developed for iron [[11], [12], [13], [14]], zinc [15,16], calcium [17], vitamins [18,19], protein [19], and calories [19,20]. To be useful on a nutrition label or to compare food items and meals, absorption and bioavailability should be considered independently from knowing host-specific factors of the consumer. Many previous prediction equations have incorporated host-specific factors. These are useful when host-specific factors are known for an individual or specific population application. When a prediction equation uses relative absorption, by comparing to a reference food or supplement (referent) as was done for calcium [17], host-specific factors do not need to be considered. However, if the relative bioavailability of the test food or supplement to a referent is different for the population or individual of interest compared with the population that was studied, as might be the case in different age groups (e.g. infants) or physiological states (e.g. pregnancy or baseline status), new evidence would be needed.

The International Life Sciences Institute (ILSI), under the leadership of the ILSI United States and Canada entity, established a Vanguard Committee to develop a research program to consider applications of prediction equations to determine nutrient bioavailability that could be applied to harmonize food and supplement databases. A major barrier to the adoption of globally harmonized nutrient reference intakes is the wide range in nutrient absorption among diets within and across regions. Furthermore, robust food composition data are lacking or insufficient in some regions to determine total nutrient intakes from consumed foods, let alone bioavailable nutrient intakes. Shortfall of vitamins and minerals may be a priority for the development of prediction equations to assist with strategies for resolving nutrients of public health concern [21]. However, prediction equations for nutrients not deemed of public health concern, as well as bioactive compounds, would also be useful in the many applications described in Table 1. These prediction equations are designed to estimate nutrient absorption or bioavailability (and ultimately bioefficacy) from foods, meals, or supplements, that is, total dietary intake, to offer many applications across various sectors. They could support the food industry with formulating products and labeling of bioavailable nutrient content. Researchers can employ these tools for targeted diet interventions, and educators and practitioners for providing dietary advice. Prediction equations also aid in assessing nutrient sustainability and availability in regional and global food systems.

**TABLE 1:** Application of nutrient absorption and bioavailability prediction equations: impact and target audience.

Applications(s)	Explanation	Impact	Interested groups
Enhanced product formulation and labeling	The ability to formulate and label the bioavailable nutrient content per serving allows for precise adjustments in labeling practices, reflecting values based on the food matrix, which can impact fortification strategies.	• Changes in nutrient labeling/serving by adding or replacing analytically determined nutrient content. • Changes in processing—fortification or refining.	Food/beverage companies; ingredient manufacturing companies
Accurate estimation of nutrient contribution	By obtaining the absorption value of nutrients in foods, integrating these into food composition databases, and applying them in assessments of diets, nutrient intake for individuals and populations can be more effectively assessed.	• Changes in dietary guidance for individuals and local populations.	Providers: medical doctors, practitioners, registered dietitian nutritionists researchers (epidemiologists) and public health institutes
Advancement in research	By establishing a common basis for comparing nutrient intake across populations, these predictive equations facilitate improvements in research methodologies.	• Changes in research methods through to compare populations. • Reduction of error (bias).	Academic; government; and industry research groups
Informed policy development	Enable the development of national and regional strategies to address nutrient deficiencies through accurate data assessment.	• Changes in research leads to informed policy decisions.	Government officials; Food industry; providers: medical doctors, practitioners, registered dietitian nutritionists, public health institutions
Sustainability	Evaluate the sustainability of nutrient resources within the global food system, providing valuable insights for long-term planning and resource management.	• Reduce ingredient waste.	Ingredient manufacturing companies; Public Health institutes, UN institutes, NGOs; food/beverage companies

**FIGURE 3:**
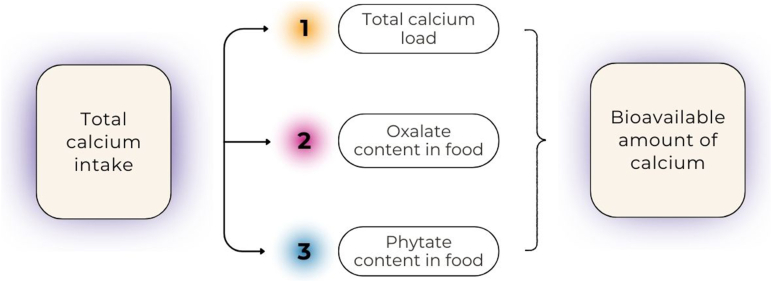
Factors used in developing the calcium absorption prediction equation, that is, the total calcium ingested from a given food and calcium absorption inhibitors oxalic acid and phytic acid.

ILSI United States and Canada convened an international working group of experts in February and March of 2024 to consider the development of nutrient absorption and/or bioavailability prediction equations. Over the next year, using working subgroups and consensus-building meetings, this framework was developed. It was recognized that individual nutrients vary in the amount and extent to which dietary factors affect their absorption. Therefore, differences in the types and levels of evidence needed are anticipated when developing absorption and/or bioavailability predictive equations for nutrients and bioactive compounds. We used the prediction equation for estimating calcium absorption of foods developed using the process in Figure 3 as a template and test case nutrient for developing prediction equations for absorption of other nutrients. The goal was to develop predictive equations that considered only factors external to the host to enable the equations to be used for food labels where the consumer is not known. This goal demands the outcome values to be determined as relative bioavailability to a reference material (e.g. food or compound) rather than as absolute values so that sources can be compared with each other for nutrient bioavailability. This will be discussed further in Methodologies for determining absorption section.

**FIGURE 4:**
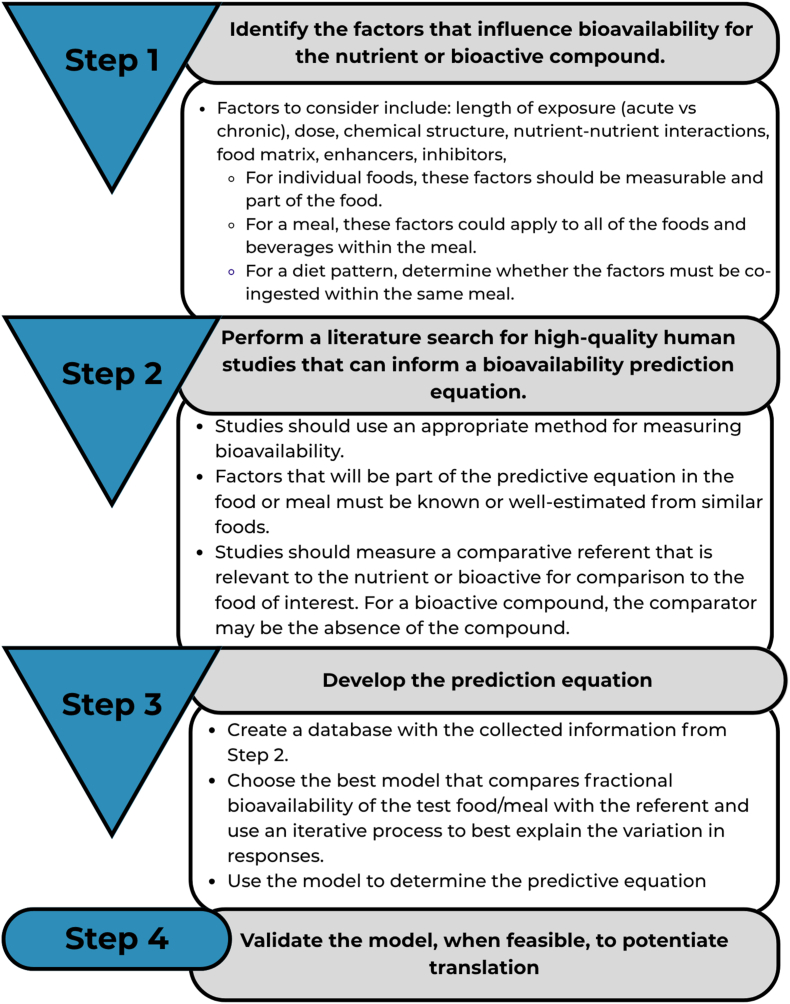
A framework illustrating a systematic approach, highlighting key considerations for expert panels or research teams in developing a nutrient absorption or bioavailability prediction equation.

The following sections outline the step-by-step process and key decisions involved in developing prediction equations for each nutrient or bioactive compound. Figure 4 provides a visual summary of the necessary steps and considerations. The proposed approach involves convening working groups for each nutrient, composed of subject matter experts, to review the steps below, make informed decisions, and collaboratively develop the prediction equations.

### Step 1: identify the factors that influence bioavailability for the nutrient or bioactive compound

**FIGURE 5:**
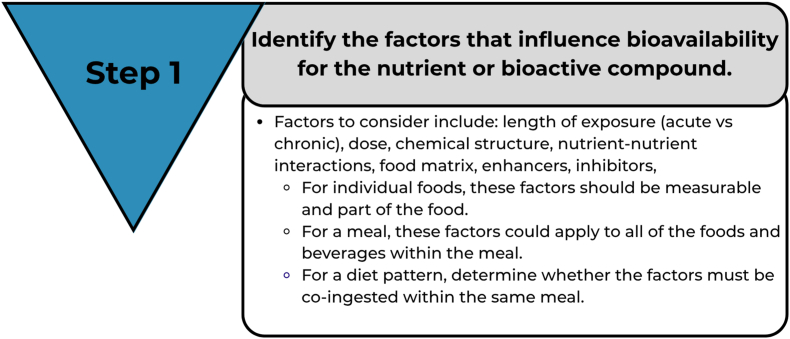
Step 1 in the framework.

The first step in the process for developing a prediction equation is to identify key factors that influence nutrient or bioactive compound bioavailability as outlined in the concept map (Figure 2). Factors to consider when deciding what information is appropriate for predictive equation development include the nature of the nutrient or compound of interest, the matrix, and the study designs used to collect the data for the desired outcome. Foods are complex mixtures of chemicals arranged in a physical environment that can influence absorption and bioavailability (Figure 5) [22].

Using calcium as an example, we discuss the influence of the food matrix. Calcium is found in different matrices in nature. In milk, two-thirds of the calcium is in the form of micellar calcium phosphate encapsulated within a protein cluster of casein micelles. Milk is one of the most concentrated and bioavailable sources of calcium. In contrast, when calcium is bound to oxalic acid, as occurs in spinach, it forms the insoluble salt, calcium oxalate, so that the relative bioavailability of calcium from spinach is only one-tenth that of calcium from milk, studied at the same calcium load [23].

Plant foods are comprised of cell walls that may encapsulate minerals even without absorption inhibitors to inhibit bioaccessibility, or substructures that may separate calcium from its inhibitors. This may explain why calcium bioavailability from soy products, which contain enough oxalate and phytic acid to completely bind all the calcium on a molar basis, is relatively high. Processing, preparation, and storage can alter these structures and complexes, changing the nutrient bioavailability. Factors that influence the bioaccessibility of nutrients and bioactive compounds will intrinsically impact the efficiency of bioconversion.

Study designs and choice of method to determine absorption influence results due to different exposures to the nutrient, different sampling schemes, whether in a fed or fasted state, sensitivities of the measurement, etc. Many studies employ tracers to follow the nutrient of interest through digestion and absorption to distinguish what comes from the test food from other sources. Much of what will be discussed below focuses on the use of tracers as the historic reference standard to measure absorption.

## Factors to Consider in the Development of Prediction Equations

### Length of exposure (acute compared with chronic)

Studies designed to measure the absorption of a nutrient or bioactive may administer the test food as a single meal or through chronic feeding to determine adaptation to exposure to the food. Both of these questions may be of interest depending on whether the food being tested is a single-source food or a staple food eaten daily compared with eaten occasionally. For the outcome of interest, a steady state may be desired, such as for determining nutrient balance. For an acute study design to determine absorption from the duodenum into the blood, giving a test meal after an overnight fast is a sufficient study length. The standardized breakfast would be designed to minimize constituents that influence the absorption of the nutrient or bioactive being tested, other than in the test food. If a tracer is being followed into the stools, controlling the diet for the duration of the gut transit time is important. If absorption occurs in the lower gut as with iron and calcium absorption enhancement by gut microbiota, 2 d or longer may be needed to monitor the tracer. Monitoring incorporation into tissues, such as iron into erythrocyte hemoglobin, can require weeks.

### Dose

The European Food and Safety Authority defines dose as “the total amount of a substance (e.g. chemical or nutrient) given to, consumed or absorbed by an individual” [24]. Because the dose “given to” and “consumed” can differ from “absorbed” by an individual, for this framework, the dose of a nutrient will specifically pertain to the amount consumed in food or supplement form in an eating occasion. The dosage can impact how much of the nutrient or bioactive is absorbed. Absorption is higher in a state of deficiency than when stores are replete for iron [25] and zinc [26]. Although actual amounts absorbed typically increase, fractional absorption declines with increasing intake, for example, magnesium [27], and zinc [28]. In contrast, water-soluble vitamins like vitamin C can be absorbed into the blood (and body) at much higher doses [29].

### Chemical structure and physical form of nutrient or bioactive

Determining the chemical structure of the nutrient, be it intrinsic (e.g. folate) or added to a food for enrichment or fortification purposes (e.g. folic acid), or present in a dietary supplement (e.g. folic acid), will be critical to establish the load. An example of the influence of chemical structure on absorption is vitamin A. A supplement with preformed vitamin A is ∼90% bioavailable, whereas the absorption of provitamin A (mostly β-carotene) is dependent on the amount of fat it is accompanied by during ingestion [30,31]. In the case of magnesium, organic forms are more readily absorbed compared with most inorganic forms (with the exception of magnesium chloride), with magnesium oxide, the most predominant form on the market, being very poorly absorbed. Another example of chemical form is iron (Fe), where heme iron is better absorbed than nonheme iron, and ferrous sulfate (FeSO_4_) and ferrous fumarate have higher bioavailability compared with elemental iron [32].

Chelators can have a positive or negative impact on absorption. Some naturally occurring chelators form insoluble salts and act as inhibitors (see next section). However, EDTA chelates with virtually every metal, and when used in food fortification, it has been shown to safely increase absorption of iron (compared with iron sulfate), zinc, and copper, especially in predominantly cereal- and legume-based diets [33,34]. Although EDTA-chelated minerals increase bioavailability by 2–3 times, the adoption of mineral chelates has been disadvantaged by higher cost (compared with other mineral forms) in combination with the food/supplement labels reporting mineral content (mg per serving) rather than the bioavailable dose.

Physical form, for example, powder compared with oil when added to foods or supplements, can also impact bioavailability. For example, fat-soluble vitamins need to be suspended in oil to be readily absorbed by the body. Furthermore, particle size plays a significant role, as micronized particles, for instance, tend to have greater bioavailability compared with larger particles. Examples of this are micronized ferric pyrophosphate and curcumin. Curcumin in its natural form has low bioavailability; however, when it is micronized, its surface area increases, which enhances absorption.

### Enhancers and inhibitors

Enhancers and inhibitors of absorption present within a food matrix or within a meal matrix should be defined. An example includes the enhancing effect of ascorbic acid (vitamin C) on iron absorption. This effect must be considered in the context of preparation and cooking methods, as these processes influence vitamin C content and subsequently impact iron absorption. Furthermore, the chemical form of the iron present in that food should be considered because the absorption-enhancing effect of vitamin C on iron varies by iron salt [35] and whether eaten acutely in a single meal or chronically.

Similarly, the effect of inhibitory compounds in a food matrix on absorption should be considered. Common inhibitors in edible crops include phenolic compounds, phytates, oxalates, tannins, lectins, enzyme inhibitors, and goitrogens, which may adversely affect the absorption of minerals, vitamins, and amino acids. Processing, that is, fermentation, germination, debranning, autoclaving, soaking, etc., can be used to reduce antinutrient contents in food products [36]. For example, during milling, the bran fraction of rice, wheat, and the endosperm in corn is removed, eliminating inhibitory compounds like phytates. This reduction in phytates would decrease their inhibitory effects on the absorption of nutrients such as zinc, iron, and, to a lesser extent, calcium [37]. The inner component of a dry bean structure, the cotyledon, holds ≤90% of the iron and zinc; however, if the cotyledon cell walls are not broken down by chewing, cooking, and milling, much of this fraction of the mineral remains inaccessible for absorption and potentially not bioavailable. An exception is the iron in soybean hulls, which is well absorbed, probably because the hull is not where the phytate is stored [38]. Matrix effects are also seen for the poor digestibility of cellulose in dark green leafy vegetables, where folate and carotenoids stored in chlorophyll are more bioavailable when plant cell walls are broken down to a greater extent [39,40]. For a prediction equation to be useful, it must have the capability to factor in both enhancers and inhibitors of nutrient absorption as is relevant. This can be extremely challenging as absorption models and food databases are limited for some nutrients and antinutrients. Quantifying enhancers and inhibitors of nutrients must also be accurately measured, which can be a challenge not only technically, but the compounds can change significantly from year to year, and location to location in the foods being produced. Thus, research models capable of defining these parameters are necessary.

### Nutrient–nutrient interactions

Commercial foods and supplements often contain several nutrients and/or bioactive compounds, and thus, they are not consumed in isolation. When 2 or more nutrients are consumed simultaneously, and 1 has a higher molar ratio, they may compete for absorption. This competitive interaction can be observed between calcium and iron, or iron and zinc [41]. In other instances, 1 nutrient may enhance the absorption of another 1 as in the case of vitamin C and iron. Dietary fiber can also play a role in altering nutrient bioavailability. For example, soluble corn fiber consumption can increase bone calcium retention [42].

### Effects of processing and/or preparation

The chemical form and matrix of enhancers and inhibitors may change through preparation of the food or integration into an enriched or fortified food or dietary supplement. Furthermore, is that food or supplement consumed in isolation or in the context of a meal? Considerations for adjustments should therefore be made for retention, gain, and loss during preparation, processing, storage, and consumption. The same factors affecting the absorption of a particular nutrient in 1 food or supplement may not be applied to that nutrient in every food or supplement, nor to other nutrients, so some assumptions may have to be made. For example, exposure to heat, oxygen, and UV light has large effects on the loss of vitamins, but not necessarily on minerals. Furthermore, cultural variability in food preparations should also be considered, as these may differ substantially compared with published literature, for example, cooking time, and time of fermentation.

Changes in a nutrient’s chemical form and properties of foods during processing and preparation can greatly alter nutrient absorption. How is the food stored until preparation, processing, and consumption? Is the food to be eaten raw? Does the preparation method alter the presence of a nutrient, for example, vitamin C is leached when cut foods are washed or stored cut for long periods of time before consumption [43]. If a food is cooked, does the cooking method alter the nutrient load in the cooked food? For example, boiling vegetables can reduce the load of water-soluble vitamins such as vitamin C. Conversely, cooking with a fat can enhance carotenoid bioavailability [44] and influence the absorption of fat-soluble vitamins. The structural form of a food may also influence the inhibitory properties of a food (see enhancers and inhibitors). Processing steps that involve maceration of cell walls may increase the amount of a nutrient that is available for absorption, or alterations to the food matrix, such as the formation of resistant starch, may influence bioaccessibility or bioavailability of associated nutrients or bioactive compounds [45].

New technologies, that is, encapsulation, nanoparticulation, and chelation, are promising for food fortification [46]. Nanoencapsulation (also called microencapsulation) encloses bioactive compounds in liquid, gaseous, or solid states within a matrix to preserve the coated substance. This technique is used to protect against heat, oxidation, and light to achieve higher levels of added nutrients in foods, that is, enrichment and fortification, and dietary supplements [47]. It has the potential to increase the delivery of an otherwise poorly absorbed bioactive or nutrient in foods and supplements.

## Bioconversion: Considerations for Estimating Bioefficacy

For some nutrients and bioactive compounds, bioconversion of bioactive food components is a critical determinant of bioavailability, representing the complex series of chemical, enzymatic, and microbial processes that hydrolyze and/or transform ingested compounds into forms that can be absorbed, metabolized, and utilized. The content of a dietary agent in food represents its maximal potential contribution to nutritional status in the host. However, inefficiency can arise, in part, due to intestinal- and liver-level bioconversions that preclude full absorption and bioavailability of dietary agents from reaching target sites where bioefficacy can be realized.

Bioconversion processes that affect bioavailability can occur independently or in a stepwise fashion in the lumen of the intestine, within enterocytes, and extending to the liver (Figure 3). Factors that are potentially involved include enzymatic hydrolysis, microbial metabolism, interactions with the food matrix (e.g. chelation), intracellular biotransformation, and hepatic first-pass metabolism. Considering these interconnected mechanisms is essential for developing a predictive framework capable of accurately modeling a nutrient or bioactive food component bioavailability and supporting evidence-based dietary recommendations to achieve optimal nutritional status. For most nutrients, such considerations are not yet methodologically possible but may be in the future.

### Bioconversion in the small intestinal lumen

Bioconversion in the small intestinal lumen represents the initial step in transforming dietary agents into absorbable forms. This process is primarily driven by chemical and enzymatic digestion and is unlikely to introduce significant variability into predictive equations of absorption for most nutrients and bioactive food components in healthy individuals, which is the focus of this predictive framework. However, it is worth noting that special considerations may be warranted in the future if the proposed predictive equation extends beyond healthy people.

Regardless of health status, polyphenols and other phytochemicals also require special attention due to their unique bioconversion pathways. In the upper gastrointestinal tract, enzymes such as lactase phloridzin hydrolase, and cytosolic β-glucosidase hydrolyze glycoside-bound polyphenols, facilitating their enterocyte uptake. The expression and activity of these enzymes are thought to exhibit interindividual variability. Furthermore, certain phytochemicals, including many polyphenols, exhibit poor absorptive ability in the small intestine, resulting in their delivery to the lower gut. In the colon, the microbiota hydrolyzes these plant-derived compounds into smaller molecular weight metabolites, some of which have known bioactivity. These metabolites can subsequently be absorbed and contribute to systemic bioefficacy. Thus, a predictive framework would need to consider estimates of uptake at the enterocyte for intact dietary agents and any of their microbiota-derived metabolites that are taken up at the enterocyte and contribute to the dietary agent’s bioefficacy. Furthermore, as rigorous and validated data become available, the predictive framework may require refinement to consider prebiotic activities of dietary patterns (e.g. high-fiber diet) on the gut microbiota that influence bioconversion of dietary agents.

### Uptake and metabolism in and beyond the enterocytes

Metabolism and trafficking within enterocytes represent a critical determinant of absorption and may be affected by host nutritional status. Host nutritional status is known to affect expression and activity of transporters and storage proteins such as with iron, zinc, and copper that can limit cellular uptake or promote intracellular sequestration of these compounds. For other nutrients, such as calcium, there is codependence of vitamin D status that influences absorption. In contrast, various phytochemicals can exhibit variability of enterocyte metabolism, which must be accounted for when predicting absorption. For example, the provitamin A carotenoid β-carotene can undergo symmetrical cleavage by β-carotene 15,15′-dioxygenase [48], yielding 2 molar equivalents of retinal. Alternatively, asymmetric cleavage may occur, producing apocarotenals that exert differential bioactivity. Xenobiotic metabolism, including phase I (e.g. hydroxylation via cytochrome P450s), II (e.g. conjugation reactions such as glucuronidation), and III (e.g. cellular efflux via p-glycoprotein) processes can also substantially influence phytochemical absorption, and with many genetic polymorphisms that not only affect dietary agent absorption but alter the form of the compound that is ultimately absorbed, which may or may not have bioactivity [49]. For example, many aglycone forms of polyphenols can undergo phase II conjugation reactions involving glucuronidation, sulfation, and/or methylation. Such bioconversions may increase their propensity for phase III efflux via P-glycoprotein or ATP binding cassette transporters to the intestinal lumen, but conjugated forms of polyphenols also appear in the circulation. Thus, a predictive framework having consumer utility will need to thoughtfully define in a dietary agent-specific manner whether only the nonbioconverted form is considered or whether to include any bioconverted forms to account for total absorption if such forms are bioactive.

Liver metabolism, which can influence bioavailability, may also need to be considered when developing predictive equations. For example, retinoids can be esterified and stored in the liver as retinyl palmitate or be converted from retinol to retinaldehyde and subsequently irreversibly transformed into retinoic acid. Predictive equations may also need to consider that provitamin A carotenoids can be bioconverted to generate 1 or 2 molar units of retinol. Although vitamin D [ergo (D2)/cholecalciferol (D3)] requires 2 separate hydroxylation reactions, 1 in the liver and 1 in the kidney, 25(OH)D only needs 1 reaction to form the bioactive 1,25-dihydroxyvitamin D, which explains the higher bioefficacy of 25(OH)D as compared with vitamin D. The FDA and Health Canada labeling requirements do not differentiate between vitamins D2 and D3. Nonessential forms of vitamin E undergo extensive xenobiotic phase I/II metabolism in the liver, bioconverting these vitamers into water-soluble metabolites, which are primarily eliminated. However, small amounts of these vitamers are secreted intact into the plasma as part of VLDL, and their water-soluble metabolites, some of which are suggested to have bioactivity, can also be observed in the circulation. In contrast, α-tocopherol, the only essential form of vitamin E, participates in these xenobiotic pathways to a lesser extent except in situations of excessive hepatic exposure of naturally occurring RRR-α-tocopherol or ingestion of synthetic or all racemic-α-tocopherol that is common in fortified foods and supplements.

In conclusion, bioconversion along the gut–liver axis determines the absorption, bioavailability, and bioefficacy of dietary compounds. Although absorption is synonymous with bioavailability for most nutrients, compounds that undergo significant hepatic metabolism—such as retinoids, vitamin D, folate, and certain phytochemicals—require additional consideration in predictive equations. The liver’s role in modifying these compounds, either by activation or deactivation, directly influences their availability for systemic circulation.

### Step 2: perform a literature search for high-quality human studies that can inform a bioavailability prediction equation

**FIGURE 6:**
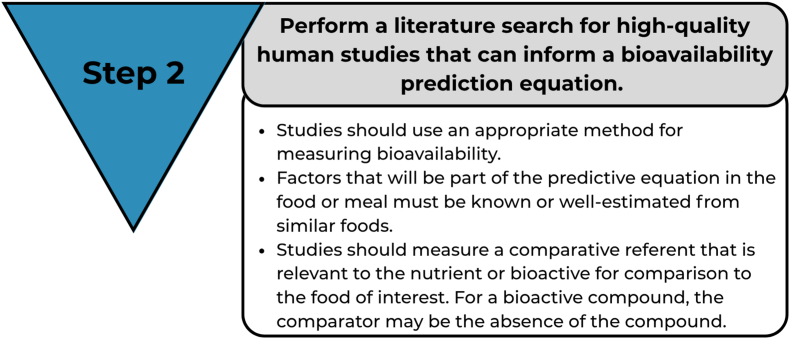
Step 2 of the framework.

After the concept map is developed that identifies factors influencing absorption, a comprehensive and systematic review of high-quality literature is needed to inform predictive equation development and to create a database. Steps 1 and 2 are iterative because the concept model is likely to be revised as the literature is reviewed and the database is being built. Studies should be conducted on human participants unless a validated method based on prior human research is already established (Figure 6).

Studies that provide useful data for building a database must be selected as appropriate for the outcome of interest. For example, it must be determined a priori to include in a database whether absorption of the nutrient of interest is relevant for a single eating occasion or adapted to chronic exposure. Mixing study designs in 1 database may confound algorithm development. When applied to a food label or a varied diet, short-term studies are appropriate. If a food is a staple, consumed daily, chronic study designs are appropriate. Whether or not adaptation occurs depends more on the food matrix than the nutrient. For example, calcium absorption is higher in the presence of some food matrices such as whey or proteins in a single meal study, but the enhancement disappears with chronic feeding, with adaptation [50,51]. In contrast, enhanced calcium absorption occurs when it is consumed with prebiotic fibers long term [52], indicating different mechanisms are involved.

Different methods for determining absorption will be described in the next section, with focus on aspects that influence absorption values, for the purpose of deciding which studies to include in the predictive equation development in Step 3. Finally, the need for studies that compare test foods to a referent will be discussed, so that relative bioavailability can be assessed.

## Methodologies for Determining Absorption

There are many study designs for evaluating the absorption of a nutrient or compound, which may have a great influence on the results. Studies that reflect absorption of the nutrient or compound of interest, as would occur for a consumer of the food, are desired. Inherent nutrient absorption can vary for any food due to many conditions. Variation in outcomes due to choice of study design decisions is an additional source of variation.

### Using isotopic tracers for determining absorption

Isotopic tracers are useful tools for measuring absorption because of the sensitivity with which they can be measured. Following the fate of the label from a food can be distinguished from other sources of the compound entering the body, during digestion, metabolism, and excretion. Following the tracer as an indicator of the nutrient or compound assumes that the tracer does not disassociate with the compound and behave differently, giving a false measure of absorption, retention, or metabolism. Dissociation of a tracer from the unlabeled portion of the nutrient does not happen with minerals where the tracer is an isotope of the mineral, but can happen with organic compounds if the tracer is an isotope of hydrogen, carbon, or nitrogen, which can be cleaved from the compound of interest. Labeling organic compounds as with deuterium or carbon isotopes to determine bioavailability has been successfully applied to vitamins A [53], K [54,55], folate [[56], [57], [58], [59]], and E [60], and polyphenols [61,62] among others.

Mathematical modeling and compartmental analysis were the data analysis approach used to calculate absorption for developing the predictive equation for calcium bioavailability [14]. Mathematical modeling is the expression of metabolism in terms of equations that describe physiological processes based on the movement of isotopic tracers. Mathematical modeling and compartmental analysis have been described for micronutrients such as calcium [63], iron [[64], [65], [66]], zinc [67], magnesium [68,69], and others. Modeling fractional absorption, rather than the amount of the nutrient or bioactive compound absorbed, allows adjustment to the amount of food being considered, for example, serving size, 100 g, etc. The amount absorbed is then the product of fractional absorption and load for that food mass.

Publicly available modeling software exists, such as WinSAAM software (www.winsaam.org). The model chosen will be based on assumptions such as the host being in steady state and the tracer load not perturbing the system. For absorption in the duodenum and determining absorption of a tracer into the blood, giving a standardized breakfast after an overnight fast is sufficient. If a tracer is being followed into the stools, controlling the diet for the duration of the gut transit time is important.

### Intrinsic compared with extrinsic labeling

Incorporating the tracer into the compound of interest is most reliable when introduced into the food as the endogenous compound being labeled. This can be done during the growth of a plant or animal food, or during the incorporation of a fortificant during the manufacture of the product. There are still many decisions for endogenously incorporating the tracer that can affect how well the tracer reflects the endogenous compound. These factors have been reviewed for intrinsic labeling of plants, which include the form of the tracer to be added, the dose, the timing and frequency of dosing, and environmental conditions such as presence of competing ions/compounds, growing plants on soil or hydroponically, temperature, pH, oxygenation, and moisture content of soil [70,71]. Digestibility of animal protein foods or plant protein foods can be measured with intrinsic labeling of uniformly 2H-labeled amino acids [72,73].

Due to the expense, labor, time required, and specialized techniques associated with intrinsic labeling, shortcuts in producing isotopic-labeled materials are attractive. The simplest technique for producing labeled foodstuffs is to extrinsically label the food by merely mixing the food with the isotope of interest. Whether or not the extrinsic label behaves as the endogenous compound should be validated. There are many examples where extrinsic labels have been found to be absorbed equivalently to intrinsic labels. For example, an intrinsic calcium isotope administered to a lactating dairy cow resulted in similar absorption from milk as an extrinsic label added to the milk [74]. The extrinsic:intrinsic calcium absorption ratio in humans was 1.03 for milk. The extrinsic:intrinsic calcium absorption ratio from eggs in rats was 0.99 [75]. Extrinsic labeling techniques often compare favorably for animal protein sources, but 1 report by Janghorbani et al. [76] found an oddly lower absorption from an extrinsic label compared with an intrinsic label of zinc from chicken and soy combinations. Extrinsic:intrinsic absorption ratios for iron, zinc, selenium, cadmium, and magnesium in rats and humans were summarized for 38 foodstuffs at the time [70]. For many foods, mineral absorption as assessed by an extrinsic label overestimated absorption from an intrinsic label by 10%–20%. There are examples of excessive overestimation of absorption by an extrinsic label when the tracer could not fully exchange with the endogenous compound, that is, the extrinsically added tracer salt is more easily ionized and absorbed compared with the endogenous compound complexed in the food. Some examples are for iron absorption in unpolished rice and calcium absorption from spinach. The extrinsic:intrinsic iron absorption ratio in humans was 1.6 for unpolished rice, 1.2 for polished rice, and 1.0 for rice flour [77]. The extrinsic:intrinsic calcium absorption ratio in rats was 2.65 for spinach compared with 0.99 for kale, a green leafy vegetable that does not accumulate oxalic acid [78]. Apparently, the unpolished grains were too dense in the case of iron, and the calcium oxalate complex in spinach was too insoluble to allow rapid isotopic exchange between the extrinsic and intrinsic labels.

Observation of extrinsic:intrinsic absorption ratios close to 1.0 did not guarantee that the extrinsic label was fully equilibrated with the intrinsic isotope. These observations simply mean that the isotopes were absorbed at an equal percentage. Further investigation is always recommended to validate the assumption of equilibration of the extrinsic label with the intrinsic isotope in the food. Research on Fe absorption has shown that equilibration of extrinsic isotopes often does not match the solubility of the intrinsic Fe; hence, equilibration was not achieved [79,80]. However, the decision to incorporate an isotope into a food intrinsically or extrinsically will depend on the purpose of the study.

### Biomarkers

A biomarker is a physiological or biochemical parameter or body characteristic that can be objectively measured to be reflective of the nutritional status of an individual for a given nutrient. To be useful, the biomarker should be indicative of the intake, metabolism, and or storage of a given nutrient. An optimal biomarker serves to objectively assess food consumption independently of consumer-reported consumption and food composition analyses. Excellent reviews have been written on the subject [31,[81], [82], [83], [84], [85]].

In general, there are 3 types of biomarkers: markers of effect or function, markers of exposure, and markers of disease state. All 3 types have a basis in nutrient intake and differ in relevance for acute compared with long-term status of a given nutrient. Biomarker measurements for nutrient status are often the nutrient itself from whole blood, plasma, serum, urine, or tissue samples, but can also include indirect physical measurements, for example, urine nitrogen for protein intake. Nutrient precursors and secondary metabolites can also serve as biomarkers. Serum 25-hydroxyvitamin D has been used as a biomarker for vitamin D bioavailability from fortified foods [86]. Challenging aspects of biomarker development and assessment are the sensitivity and specificity of the biomarker, and whether the marker represents acute or long-term nutritional status. Scientists are striving for new and improved biomarkers to evaluate nutritional status and disease states.

Biomarkers may be determined on samples collected at a single timepoint or serially over time. When isotopic tracers are not practical, such as when there is no suitable tracer for a nutrient or when measurements are sufficiently sensitive, AUC of a biomarker of exposure or status may be used. This approach is not advisable when the test load must be so large that it perturbs normal metabolism of the compound or saturates transporters to be measured. This may relate to the measurement sensitivity or to the background level of the compound, especially when the nutrient is homeostatically controlled. Acute appearance of the nutrient from the test meal into the blood has been used to estimate bioavailability of potassium [87], iron [88], and vitamin K [89]. In contrast, for vitamin D, the AUC of the status biomarker, serum 25-(OH)-D assessed weekly over 11 wk has been used to determine bioavailability from fortified orange juice [86]. Measurement of metabolites has become so sensitive that measuring bioavailability of unlabeled compounds may approximate assessment of labeled compounds, as was the case for phylloquinone [vitamin K(1)] using HPLC/mass spectrometry with atmospheric pressure chemical ionization [90]. However, the response of plasma concentrations of the compound also determines feasibility. For example, plasma retinol is under homeostatic control of the liver, and therefore, it is not a very good response marker in the vitamin A sufficient range. Inflammatory processes can also disturb biomarker concentrations.

### Slope ratio assay

Slope ratio assays, sometimes called depletion–repletion studies, have been used to determine relative absorption to a reference compound. The slope of the outcome of interest plotted against the dose of the compound of interest is assigned a value of 100%. The slope of the test compound is compared with the slope of the reference compound, which can be > or < 100% depending on whether the test compound is absorbed better or worse than the reference compound. Selection of the outcome is better if it relates to the function of the nutrient or compound. Growth has been used, but growth is not specific to any 1 nutrient. Examples of better outcome measures that relate to specific functions associated with the nutrient include hemoglobin regeneration for iron, tibia zinc content for zinc, xanthin dehydrogenase/oxidase for molybdenum, and glutathione peroxidase for selenium. This method is better for salts used as supplements or fortificants than for nutrients from foods as increasing the amount of nutrient from the food also increases the amount of other components in the food, which may confound the measurement specific for the nutrient or compound of interest.

### Balance methods

Balance studies involve a controlled diet and complete collection of urine and feces after equilibration to the diet. Net absorption is determined from intake minus excretion in feces. Net retention is calculated as intake minus excretion. Balance studies are sufficiently sensitive, when rigorously controlled, to distinguish large differences when comparing 2 or more interventions. However, balance methods are much less sensitive than methods employing isotopic tracers. Balance studies may be useful if the nutrient is not produced by bacteria in the colon, as is the case for folate and vitamin K2.

### Preclinical testing

In vitro and animal studies are useful to do prescreening before conducting expensive human studies, but they are not typically a useful source of data for predictive equation development. In vitro trials generally can only mimic a certain step in the absorption process, such as uptake into the enterocyte, or solubility at gastric or intestinal conditions. A model for Fe absorption known as the Caco-2 Cell Bioassay is one of the most thoroughly validated examples of an in vitro model widely applied for predicting human Fe absorption [[91], [92], [93]]. Another example of a validated animal model is the pig for determining true ileal digestibility of amino acids [94]. This model is especially important when it is impractical to study healthy humans.

Animal models have the distinct advantage of allowing protocols that can be more invasive, thus helping define mechanisms of action. However, physiological differences in animals compared with humans can also negate the usefulness of an animal model. For example, piglets were once thought to be excellent models for iron absorption studies; yet, unlike humans, piglets are anemic at birth and require intramuscular Fe injections for optimal health [95]. Rodents are significantly more efficient at absorbing Fe relative to humans [96,97]. If correction equations can be generated, the model can still be useful [98]. Poultry have been shown to be an excellent model for human Fe nutrition [92].

Studies used for predictive equation development should be conducted in human participants; however, when applied properly, preclinical research tools can reduce the need for some human trials and provide more cost-efficient refinement of experimental objectives for the human trials that are conducted. The future is promising for alternative models, but they must be rigorously validated. Research is needed to improve and validate models such as organoids (e.g. enteroids) and animal models where key determinants of bioavailability have been humanized (e.g. mice with humanized transporters, xenobiotic regulators, etc.). Some animal models (e.g. pig) also allow for more direct measures (e.g. true absorption of water-soluble compounds sampled from the portal vein).

## Importance and Need for Using a Referent

Given the goal of this framework, that is, for nutrient absorption prediction equations to be based on knowledge of the food without knowledge of the consumer, we recommend using a referent food or nutrient for comparison. A standardized portion of white bread has been the referent for measuring the amount of carbohydrate taken as glucose in foods and diets [99]. For developing a recent prediction equation for determining calcium absorption from foods, milk or calcium carbonate was used as the referent [17], meaning that only studies that compared calcium absorption from a food or beverage and one of these common sources of dietary calcium in the same participants were included. The observed calcium absorption was converted into a calcium absorption index, that is, the ratio of calcium absorption from the test source to the referent studied at the same calcium load in the test meal, for use in model development of fractional calcium absorption considering calcium load and absorption inhibitors. This approach assumes that all the physiological factors of the consumers that can influence absorption such as age, state of growth, gut health, status of the nutrient being studied, or related factors such as vitamin D status for calcium, are negated if both the food being tested and the referent were studied while these factors were held constant. This is reasonable if the food and referent are studied without an appreciable lapse of time, unless there is evidence to the contrary. For example, a meta-analysis and systematic review showed that vitamin D3 is more bioavailable than vitamin D2, except in those deficient with a baseline 25(OH)D of <50 nmol/L and/or BMI >25 kg/m^2^ [100].

Advantages of using a referent are strong. Often, the physiological factors of the consumer are not known or cannot be measured to make adjustments for the comparison. In the case of calcium, calcium status may have a small influence on absorption [101], but there are no good calcium status indicators [102]. Few nutrients have status indicators, such as iron, where serum ferritin has been used in prediction equations for iron absorption [13]. Without a referent, it is difficult to compare the relative absorption of a nutrient. In the development of the predictive equation for calcium absorption, data from a very important study comparing calcium absorption from tortillas made from genetically modified low-phytate maize and the higher phytate wild type could not be used due to a lack of a referent. Mean fractional absorption from tortillas providing 140 mg calcium prepared from the low-phytate maize was 0.50 ± 0.03 compared with a mean of 0.35 ± 0.07 from the wild-type maize (*P* = 0.01) [103]. These values were logarithmically different from the predictive equation and could not be explained by phytate or oxalate content. Without a referent, it was impossible to adjust for the physiological variability in the host. Papers to be used in a predictive equation would be downgraded if a referent is not included.

The disadvantages of using a referent are also significant. Few studies are available for many of the nutrients of interest that compare absorption with a referent. Furthermore, the approach assumes that relative absorption between the food and the referent is constant across populations. This may not be true, and there is little evidence to answer the question. Lastly, the choice of the referent may be obvious for some nutrients, such as calcium, but less obvious for many others where there is no single or dominant source in the diet. In this case, the group developing the prediction equation should select a referent to guide subsequent research. Nutrients that have referent-supported data become more attractive for developing predictive equations and will serve as examples for future equation development.

### Step 3: develop the bioavailability prediction equation

**FIGURE 7:**
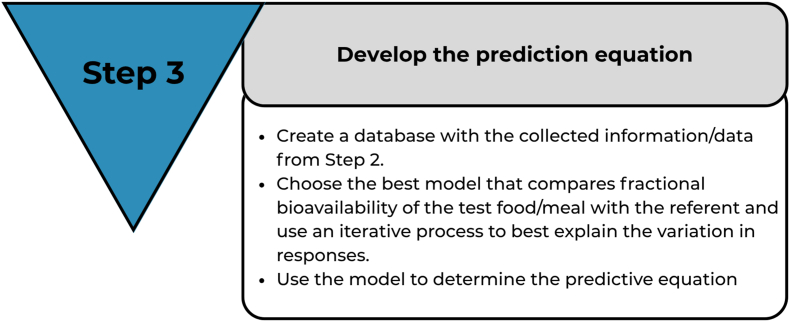
Step 3 of the framework.

In step 1, factors that influence absorption of the nutrient being studied and the nature of the relationship between each of these factors and absorption were identified. In step 2, relevant data were extracted from the literature, and the database was developed. In step 3, the predictive equation will be developed (Figure 7).

## Development of Bioavailability Prediction Equations

Having complete data on all needed variables is important to eliminate omitted variable bias and to ensure that the predictive equation accounts for as much variability in absorption as possible. This will ensure strong performance for future predictions. However, there is also the need to avoid adding too many predictors to the predictive equation, which can result in overfitting it. Where there are too many predictors, an appropriate variable selection approach should be used to minimize risk of overfitting. Another important data consideration is the source of the data. Although many predictive equations are built using secondary data, intentional data collection to meet model requirements will provide the best quality predictive equation by ensuring that all necessary variables are considered. This will also ensure that the data are representative of the target population. Consideration must also be given to the sample size, which may vary depending on factors such as the variance in absorption, the number of explanatory variables, and the type of model. To develop an ideal predictive equation, absorption and dietary intake data must be obtained using methods considered to be accurate. Data must also be inspected visually for outliers that can affect data fitting. For example, if human absorption trials using poorly equilibrated isotopes are part of the prediction equation development, then the accuracy of the equation will certainly be affected.

Several approaches to developing a predictive equation will be discussed. We begin with an example of developing a calcium bioavailability equation, then discuss regression more broadly.

### Developing a predictive equation for calcium

**FIGURE 8:**
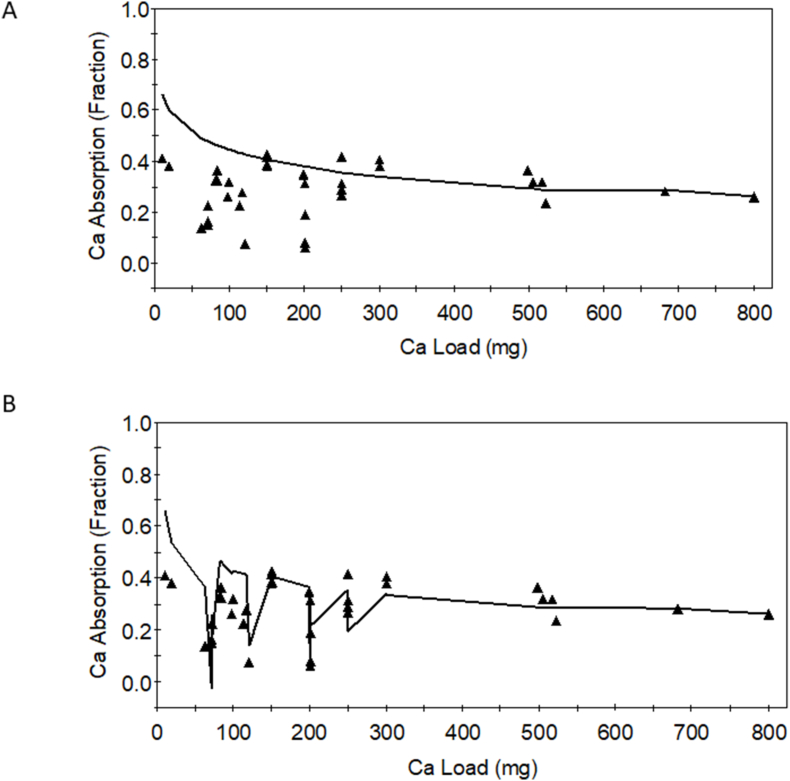
Calcium absorption as a function of calcium load (A) and as a function of calcium load, oxalate, and phytate (B) [17].

The development of the calcium bioavailability predictive equation [17] began with identifying the key factors that influence calcium absorption (Figure 3). Data were extracted from human studies using calcium isotopes to determine absorption from single meals and those that had a referent of milk or calcium carbonate. This predictive equation predicts relative calcium bioavailability for acute exposure to the test food and not for adaptation with chronic feeding. This equation is the most useful for applications such as food labeling, assessing nutrient adequacy in individuals or populations, and developing fortification strategies. The first predictive equation built used only calcium load as a factor (Figure 8A) [17]. It was clear from observing the fit of the data that the fit was poor. The next predictive equation used calcium load and phytate, and oxalate loads (Figure 8B). The fit of the predictive equation improved from explaining ∼1% of the variation in fractional calcium absorption using calcium alone to explaining 45% in the model with calcium load, oxalate, and phytate. We were able to readily observe when data were added to the predictive equation that did not fit, for example, data without a referent, with absorption values that were extremely high.

The predictive equation will always include the load of the nutrient or bioactive being considered. Additional parameters such as known inhibitors can be added to the predictive equation to determine their contributions to fitting the data. In addition to the underlying assumptions of a predictive equation, an important criterion of equation development is how well the predictive equation fits the data. The predictive equation can be optimized by allowing the fractions of the inhibitors to vary and minimizing the squared residuals between observed data and model-calculated values, using the least squares fitting routine in WinSAAM. The variation in data explained by the predictive equation can be determined with standard statistical approaches such as the Pearson function in Excel.

In regression analysis, absorption is made the outcome variable with dietary factors (inhibitors and enhancers) as predictors or regressors. There are diverse types of regression models, ranging from simple linear regression with 1 predictor to more complex ones such as mixed-effect models, which account for random-effects variables [13]. The type of regression model used depends on the nature of the data. Even with the regression models, dietary predictors can still be treated qualitatively as binary variables (either present or absent from the diet). An example of this type of model, which compared absorption from foods to a referent, was developed by Conway et al. [104] for predicting nonheme iron absorption. In this model, log-transformed nonheme iron absorption is estimated from a regression model that uses the presence or absence of dietary factors such as fruit juice, animal tissue, beans/lentils, whole grain cereals, tea, dairy, cheese, and soya. Each dietary factor in this model is treated as a dummy variable and takes a binary value of 0 or 1 for absent or present, respectively. However, most regression-based models tend to include dietary predictors as continuous variables.

When a regression model is used, the beta coefficients should appropriately represent the nature of the associations between each dietary predictor and absorption. It is not appropriate to have a positive beta value where there is a known inverse association between the predictor and absorption. Also, the assumptions that underlie regression analysis must be met. For linear regression, *1*) there is a linear relationship between each independent predictor and the population mean of absorption (as outcome variable) when other predictors are held constant (linearity); *2*) the error terms should be independent of one another (independence); *3*) the error terms are normally distributed with a mean of zero (normality); and *4*) the error terms should have equal variance (equality of variance or homoscedasticity) [105]. Nonlinear regression analysis requires the same assumptions, except that the relationship between the predictor variable and the outcome (absorption) must follow the assumed nonlinear curve instead of a linear relationship. Unmet assumptions can lead to inaccuracies in prediction. Diagnostic plots such as residual plots and normality plots should be used to evaluate the regression model for violation of assumption. The use of transformations can help to address some violations. Common transformation methods include log transformation and power transformation. A regression model should also be assessed for goodness of fit. This evaluates how well the model fits the data and can be done using the coefficient of determination (*R*-squared) or by evaluating the residuals. *R*-squared indicates the proportion/percentage of variance or variability in the outcome variable explained by the model. A more robust form of *R*-squared, the adjusted *R*-squared adjusts for the number of parameters in the model. There is no consensus on cut-off values for *R*-squared as this depends on what is being predicted. A good practice is to compare values with previously reported values for similar models for the same nutrient. The model should also be validated using an independent dataset to determine its performance in predicting absorption.

### Qualitative approaches

In developing predictive equations, some authors have used qualitative approaches. The qualitative approach considers the presence or absence of factors that influence the absorption of the selected nutrient. An example of a qualitative model is the FAO/WHO model for assessing iron absorption from diets, in which an absorption value of 5% or 10% is assigned to diets from lower-income countries and 12% or 15% assigned to higher-income countries diets [4]. The strength of this approach is its simplicity. The qualitative models are easy to apply and typically require less data. The major limitation of this approach is that even among similar dietary patterns, the quantity of inhibitors and enhancers consumed may differ significantly among populations and geographical areas. This approach does not consider such differences in intake but rather generalizes dietary patterns, resulting in the same absorption values being assigned to diets that might have significant differences in composition. This limitation is partially addressed by using the semiquantitative approach, in which cut-offs for the intake of the different dietary factors are used to determine the absorption of the nutrient. An example of such a model was used by Singer et al. [106] to estimate nonheme iron bioavailability from meals. For this model, meals that contain <24 mg vitamin C or <6 g of protein from meat-fish-poultry are classified as low bioavailability meals, meals with >25 mg but <75 mg vitamin C or >6 g but <18 g of protein from meat-fish poultry are classified as medium bioavailability and meals with >75 mg vitamin C or >18 g of protein from meat-fish poultry or 25–75 mg vitamin C plus 6–18 g of protein from meat-fish poultry are considered high bioavailability meals. In both the qualitative and semiquantitative approaches, the estimated bioavailability takes on 1 of a few prespecified values rather than a continuous estimate, which is another limitation. Qualitative models also often rely on expert judgment that may increase risk of biases. This method is thus useful when there are limited data, or insufficient data to develop models from numerical analysis.

## Validate the Predictive Equation

Step 4, the concluding stage of the framework, focuses on validating the predictive equation when feasible and enhancing its applicability for practical implementation. The typical approach to validating an equation is to see how well data fit the equation from datasets not used to develop the equation (independent dataset) but that use experimental designs and endpoints consistent with the datasets that were used to develop the equation [107]. Preserving an independent dataset from equation development will be difficult to achieve for many nutrients and bioactive with existing literature.

## Development of Predictive Equations for Specific Populations

Application of prediction equations for populations or individuals may be modified for populations where host factors are known. If a fortification or supplementation strategy is being designed for a population known to be deficient in the target nutrient, a predictive equation that includes an adjustment specific to that population could be developed, or a safety margin added. Deficiencies of a nutrient may influence the relative absorption of the nutrient as has been shown for iron [11,12]. Furthermore, bioavailability and bioefficacy should not be confused: bioavailability per se may not be compromised, but bioefficacy of a nutrient, once ingested and absorbed, may be compromised due to nutrient status, disease state, or life stage. For example, iodine bioavailability, in the form of iodine uptake, is affected by the degree of deficiency of the host. If the host thyroid is more replete, less iodine will be taken from the circulation than if the host thyroid is deficient. Disease states may also change nutrient absorption, such as obesity, which leads to chronic inflammation, thereby increasing hepcidin production and leading to less iron absorption. The cellular status of a nutrient can also dictate absorption. For example, vitamin A cellular status will dictate how much provitamin A is converted to vitamin A. If the cellular stores are plentiful, the intact provitamin A will be the form that is absorbed and stored [108,109].

Life stage is another important factor, with nutrient uptake affected in times of increased nutrient need, such as pregnancy, lactation, and infancy, or through physiological changes during certain life stages such as suboptimal digestion in older age. Different models may be needed for different life stages for some nutrients. Populations may have known food intolerances such as lactose that can interfere with the absorption of nutrients [110] or genetic variants that can influence the absorption of nutrients [111] and bioactive compounds [111].

In conclusion, nutrient absorption prediction equations can improve the evaluation of the nutritional value of a food, meal, or diet and the relationship of this improved accuracy to health. For example, the calcium bioavailability prediction equation was applied to NHANES data on food intake to show trends in bioavailable calcium intake and its relationship to fracture incidence in the United States [112]. An individual who wishes to use an application of the predictive equation to estimate bioavailability may have a disease state or condition with a known effect on nutrient absorption, or be taking medications that interact with nutrients. They can adjust their recommendations accordingly.

Often, the relative absorption to the referent for that individual will remain the same. Here, an international group of experts proposes a framework to guide expert panels in developing nutrient absorption prediction equations for individual nutrients and bioactive compounds. Each nutrient will need to be evaluated for matrix factors that influence absorption. For many nutrients, additional research is needed to form a meaningful absorption prediction equation. However, this international panel of experts views such efforts as critical for informing key knowledge gaps that can be addressed experimentally utilizing a much-needed framework of nutrient absorption that can prevent malnutrition and promote health across the lifespan.

## Author contributions

The authors’ responsibilities were as follows – RSB, TK: designed the final figure; and all authors: contributed to writing sections, revising drafts, and reviewing and approving the final manuscript.

## Funding

This project was funded by The International Life Sciences Institute (ILSI), U.S. & Canada, a nonprofit scientific organization. ILSI, US & Canada paid for publication fees. The project was led and managed by ILSI U.S. & Canada staff, who are listed as authors on this paper. ILSI US & Canada receives its support primarily from industry.

## Conflict of interest

CW reports a relationship with The International Life Sciences Institute that includes: travel reimbursement. CW is on the ILSI Global Board and The Chair of the Science Board for ILSI US & Canada. CW chairs the Application of Globally Harmonized Nutrient ILSI US & Canada Committee; RSB is the current editor-in-chief of Nutrition Research, serves as a scientific advisor for Gem Health, Inc. (Venice, CA, USA) and the Hass Avocado Board, and has received research grants and/or honoraria for scientific review activities from the NIH, USDA, American Egg Board/Egg Nutrition Center, National Dairy Council within the past 5 years; AF is employed by Fonterra and has an appointment with the Sustainable Nutrition Initiative; MIM is a member of the ILSI US & Canada Science Board and on the ILSI US & Canada Application of Globally Harmonized Nutrient Committee. During the past 3 years, MIM has had financial agreements with the Council for Responsible Nutrition; Fatty Acid Research Institute; International Life Sciences Institute, North America; McCormick Science Institute; and the WHO; TCW is the Editor-in-chief of the Journal of Dietary Supplements, Deputy Editor-in-chief of the Journal of the American Nutrition Association, and Nutrition Section Editor of Annals of Medicine. TW receives royalties from the Academy of Nutrition and Dietetics for his editorship of the Health Professionals Guide for Dietary Supplements. TW has received competitive unrestricted research grants from the American Pulse Association, Balchem, Corp., Egg Nutrition Center, Florida Citrus, National Dairy Council, National Pork Board, Nestle Health Sciences, New Capstone, Inc., Oak Ridge Institute for Science and Education, The Kraft Heinz Company, & Simply Good Foods Company, and Reach Global Strategies. TW has received scientific consulting fees from the Academy of Nutrition and Dietetics, Haleon, National Pork Board, and & The Beer Institute. TW has received speaker honoraria from the American Society for Nutrition, Berry Health Benefits Symposium, International Food Additives Council, Amazentis, Tea Institute, Forbes Health, National Dairy Council, Balchem, Corp., San Diego State University, and the University of Missouri. TW is a member of the scientific advisory boards for Deerland Probiotics & Enzymes, Forbes Health Advisory Board, and Haleon. TW is an unpaid senior fellow at the Center for Magnesium Education & Research. TW has an up-to-date ICMJE disclosure of interest statement available on his website www.drtaylorwallace.com. RSB is the current editor-in-chief of Nutrition Research, serves as a scientific advisor for Gem Health, Inc. (Venice, CA, USA) and the Hass Avocado Board, and has received research grants and/or honoraria for scientific review activities from the NIH, USDA, American Egg Board/Egg Nutrition Center, National Dairy Council within the past 5 years. EU is part of the ILSI Global Board and a Scientific Director of ILSI-SE Asia and a Co-Chairperson of the Nutrient Recommendations Working Group, a project under ILSI-SEA. The other authors report no conflict of interest. If there are other authors, they declare that they have no known competing financial interests or personal relationships that could have appeared to influence the work reported in this paper.
